# Bovine natural antibody IgM inhibits the binding of human norovirus protruding domain to its HBGA receptors

**DOI:** 10.1002/2211-5463.13450

**Published:** 2022-06-16

**Authors:** Qi Han, Zhaolei Xue, Ming Tan, Likai Wang, Huiling Chen, Ran Zhang

**Affiliations:** ^1^ State Key Laboratory of Agrobiotechnology, College of Biological Sciences China Agricultural University Beijing China; ^2^ Division of Infectious Diseases Cincinnati Children's Hospital Medical Center OH USA; ^3^ Department of Pediatrics University of Cincinnati College of Medicine OH USA

**Keywords:** antiviral, bovine colostrum, HBGA, human norovirus, IgM, natural antibody

## Abstract

Human norovirus (HuNoV) is the primary viral pathogen that causes acute gastroenteritis (AGE) in humans. The protruding (P) domain of HuNoV interacts with cell surface histo‐blood group antigens (HBGAs) to initiate infection. Owing to the lack of an effective *in vitro* culture method and a robust animal model, our understanding of HuNoVs is limited, and as a result, there are no commercial vaccines or antivirals available at present against the virus. In an attempt to develop a preventative measure, we previously identified that bovine colostrum (bCM) contains functional factors that inhibit the binding of HuNoV P domain to its HBGA receptors. In this study, a candidate functional factor in bCM was identified as immunoglobulin M (IgM) using mass spectrometry, followed by database comparison. The natural antibody IgM was further verified to be a functional protein that inhibited HuNoV P protein binding to HBGA receptors through receptor‐binding inhibition experiments using bCM, commercial IgM, and fetal bovine serum. Our findings provide a foundation for future development of natural IgM into an antiviral drug, which may help to prevent and/or treat HuNoV infection.

## Graphical abstract

The natural antibody IgM was verified to be a functional protein that inhibited HuNoV P protein binding to HBGA receptors through receptor‐binding inhibition experiments using bCM, commercial IgM and fetal bovine serum.

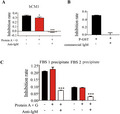

**Abbreviations**
2D PAGE
: two‐dimensional polyacrylamide gel electrophoresis
AGE
: acute gastroenteritis
bCM
: bovine colostrum
bCM1
: bCM sample 1
Co‐IP
: co‐immunoprecipitation
ELISA
: enzyme linked immunosorbent assay
FBS
: fetal bovine serum
HBGAs
: histo‐blood group antigens
HuNoVs
: Human noroviruses
IEF
: isoelectric focusing
IgM
: immunoglobulin M
IP
: immunoprecipitation
IPG
: immobilized pH gradient
nIgM
: natural IgM
PI
: isoelectric point
pIgR
: polymeric immunoglobulin receptor
SDs
: standard deviations

Human noroviruses (HuNoVs) are the leading viral cause of infectious acute gastroenteritis (AGE), resulting in substantial global morbidity and mortality [1, 2]. While HuNoVs are genetically diverse, GII.4 (genogroup II, genotype 4) is the most prevalent genotype. GII.4 viruses are known for their stronger mutative and infectious ability, which allow them quickly mutating to escape host immunity and cause large‐scale outbreaks globally [3, 4]. HuNoVs are members of the *Norovirus* genus in the *Caliciviridae* family, which are a group of non‐enveloped RNA viruses with icosahedral capsids that encapsulate RNA genomes [5]. The capsid protein VP1 that constitutes the viral capsid consists of two main domains, the N‐terminal shell (S) domain and C‐terminal protruding (P) domain. The S domain forms the interior shell, which wraps the viral RNA, whereas the P domain builds exterior protrusions extending from the inner shell of the capsid. HuNoV infection initiates on interactions of the capsid P domain with histo‐blood group antigens (HBGAs) on host cell surface [6, 7, 8, 9]. Owing to the lack of an effective cell culture system and a robust animal model [10, 11], much HuNoV research remains in the stages of an *in vitro* experiment and our understanding on HuNoV remains limited. For example, an enzyme‐linked immunosorbent assay (ELISA)‐based binding and blocking assay using recombinant capsid proteins as HuNoV model and saliva samples as HBGA sources has been developed [12] and used to surrogate neutralization assay [13] to evaluate vaccine and antivirals. Currently, there are no licensed antiviral drugs or vaccines against HuNoVs [14].

Milk, especially bovine colostrum (bCM), is rich in antibodies and immune protective factors, such as immunoglobulins [15], lactoferrin [16], complement proteins, oligonucleotides, chitosan, lysozyme, and lactoperoxidase [17]; thus, bCM has been used to treat acute diarrhea and other gastrointestinal infections [18, 19, 20]. In a previous study [21], we showed that two components of a bCM sample 1 (bCM1), designated as component 1 and component 2, respectively, after progressive affinity chromatography (to remove IgG, anion exchange chromatography, and gel filtration chromatography, which contained an 84‐kDa protein [isoelectric point (PI) ~ 5.5], could inhibit GII.4 HuNoV P protein–HBGA interactions.

In this study, we conducted an in‐depth analysis to identify the bCM protein that blocks norovirus P protein–receptor interactions. We found that IgM, a natural antibody in bCM, is a functional protein that competitively binds to the GII.4 HuNoV P protein, thus hindering its binding to HBGA receptors and potentially inhibiting HuNoV infection. Therefore, our study provides a new foundation for the use of IgM in bCM in preventing HuNoV infection.

## Materials and methods

### Bovine colostrum samples preparation

Bovine colostrum (bCM) samples were collected from Holstein cows on day 3 after parturition or prolactin treatment. The milk samples were stored at −80 °C.

### Water precipitation of bovine colostrum

IgM proteins in bCM were precipitated by water dialysis. Specifically, the bCM samples were dialyzed twice against ultrapure water at 4 °C for 3 h. Supernatants and precipitates (resuspended in distilled water) were recovered separately and stored at 4 °C.

### Production of the HuNoV P‐GST fusion protein

The P‐GST fusion protein containing the receptor‐binding domain of the GII.4 HuNoV VA378 strain (GenBank access #: GenBank: AY038600.3) was made as described previously [21, 22]. This protein has been shown to self‐assemble into large complexes with authentic binding ability to HBGA ligands [22] and thus suitable to be used in this study.

### Blocking assays against the binding of HuNoV P protein to HBGA receptors

This assay was performed as previously described [21]. Briefly, saliva sample (OH39) with known HBGA types from our lab collection was positive for H type 1 (H1), H2, and Lewis y (Le^y^), but negative for Le^a^ and Le^x^ antigens [23]. The saliva sample was collected from an American healthy adult volunteer without collecting personal information during a previous study that was approved by the Institutional Review Board of Cincinnati Children's Hospital Medical Center [23]. This saliva sample was diluted with PBS (1 : 1000) and coated on a microtiter plate overnight at 4 °C. On the next day, the plated was blocked with 5% nonfat milk in PBST (PBS, pH 7.4, with 0.5% tween‐20). Fifty microlitre of 2 μg·mL^−1^ GII.4 HuNoV (VA398) P‐GST fusion protein were mixed with the tested samples and incubated at 37 °C for 1 h. The mixture was then added to the plate. Subsequently, the bound P‐GST fusion protein was detected using a rabbit anti‐GST polyclonal antibody (1 : 5000). The bound antibody was detected using a horse‐radish peroxidase (HRP)‐conjugated secondary antibody (1 : 10 000). The binding signal was defined as the optical density (OD) signal and was measured at 450 nm (OD_450_). The inhibition rate of each sample was calculated using the following formula:

$$\begin{array}{ll} \text{Inhibition rate} & =\left({\mathrm{OD}}_{\text{unblocked control}}-{\mathrm{OD}}_{\text{blocked test}}\right)\\ & \;/\left({\mathrm{OD}}_{\text{unblocked control}}-{\mathrm{OD}}_{\text{negative control}}\right)\times 100\%. \end{array}$$

The OD_unblocked control_ was the OD of the binding between the saliva sample and P‐GST protein without mixing with the tested sample, while the OD_negative control_ was the OD of the binding signal of the saliva sample to GST only.

### Two‐dimensional polyacrylamide gel electrophoresis (2D PAGE) and silver stain

Two components of bCM sample 1 (bCM1) were analyzed by 2D PAGE as described previously [21]. Briefly, samples containing 0.5–10 μg proteins were loaded to immobilized pH gradient (IPG) strips (7 cm, pH 4–7 or pH 3–10). The first‐dimensional isoelectric focusing (IEF) was run using Etan IPG phor3, and the second‐dimension SDS/PAGE was run using a regular electrophoresis system (Bio‐RAD, Hercules, CA, USA), and protein spots were visualized by silver stain (Bytotime, Shanghai, China) according to the manufacturer's instruction. Images of the stained gel were taken by Gel Logic 212 (Kodak, Rochester, NY, USA).

### Removal of IgM from bovine colostrum samples by immunoprecipitation (IP)

Removal of IgM from bCM samples was accomplished by an IP approach using excess protein A + G agarose (Beyotime) and a rabbit anti‐bovine‐IgM antibody (Rab‐bIgM; Rockland, PA, USA). Briefly, 200 μL protein A + G agarose was incubated with 1 mg Rab‐bIgM overnight at 24 °C with an up and down rotation; fluid was discarded after 1 min of centrifugation at 500 × ***g***, and the agarose was washed with PBST three times. Samples (10 μL bCM or FBS, or 200 μL chromatography fractions) were incubated with protein A + G agarose with or without Rab‐bIgM at room temperature for 1 h, and the total volume was adjusted to 500 μL using PBS. Subsequently, the fluid fraction was recovered and stored at 4 °C. The agarose was washed three times and stored at 4 °C.

### Western blot analysis

An equal volume of 5× loading buffer was mixed with the samples before and after treatment, which were boiled for 10 min. After separation by 10% SDS/PAGE, the proteins were transferred to a 0.45 mm PVDF membrane and blocked for 1 h with 5% nonfat dry milk at room temperature. Then, the membrane was incubated overnight at 4 °C with HRP‐conjugated polyclonal rabbit anti‐bovine IgM (1 : 1000 dilution; Rockland, PA, USA). After incubation with the secondary antibody for another hour, the membranes were developed with SuperSignal™ West Pico PLUS Chemiluminescent Substrate (Thermo, Waltham, MA, USA).

### Assay for IgM binding to HuNoV P protein

ELISA‐based binding assay to test IgM and GII.4 HuNoV P protein interaction was performed. Pure bovine IgM (Rockland) or IgM‐containing fractions obtained from dialysis of bCM were coated onto microplates overnight at 4 °C. After blocking with 5% nonfat milk in PBST, P‐GST, GST, or other indicated materials were added and incubated at 37 °C for 1 h. Corresponding primary and secondary antibodies were used to detect bound proteins. The OD_450_ was measured as previously described [21].

### Ethics approval

All procedures involving animals were approved by the Laboratory Animal Welfare and Animal Experiment Ethics Review Committee of China Agricultural University (No. AW42302202‐3‐1) and in accordance with the NIH Guide for the Care and Use of Laboratory Animals.

### Statistical analysis of data

Data are expressed as the mean ± standard deviations (SDs) and were analyzed using graphpad prism software 7.00 (GraphPad, San Diego, CA, USA). Statistical significance among data groups was calculated by the *t*‐test and was considered statistically significant when *P* values < 0.05. The data for molecular protein weights and isoelectric point (PI) values were analyzed using UniProt.

## Results

### IgM identified as a candidate protein by mass spectrometry

As per our previous experiment, bCM sample 1 (bCM1) comprised of two components [21]. Component 1 of bCM1, which is rich in functional proteins, was analyzed by mass spectrometry, where 48 proteins were detected. The isoelectric point (PI) values and molecular weights of all the detected proteins were determined using the UniProt database. According to the mass spectrometry results, the highest score was human keratin, which was contaminated during the operation. Serum albumin and IgG proteins, which are abundant in regular milk and are unlikely to be target proteins [19]. The next proteins based on the scores were IgM and JUP (Table 1). The JUP is mainly present on the cell membrane and is a protein secreted in unconventional milk, most likely a component of exfoliated cells. After a literature review, we found IgM is the second most abundant antibody in bCM with ability to activate complement immune responses, inhibit pathogen‐receptor binding, agglutinate pathogens, and neutralize toxins and viruses [24, 25, 26, 27] (see Discussion for more details) and thus set off to test gM first.

**Table 1:** Main mass spectrometry (MS) results in component 1. The source of the IgM is from *Suncus murinus*, and the constant region (C region) of the IgM heavy chain possesses a molecular weight (MW) smaller than the full‐length murine heavy chain. According to published results, the IgM heavy chain is located at a position approximately 80 kDa on a reduced SDS/PAGE gel. Score: protein matching score; higher score indicates higher reliability. PI, isoelectric point.

Accession no. (Uniprot)	Protein name	Score	Mass	PI
P04264	Keratin, type II cytoskeletal 1	3453	66 170	8.15
P35908	Keratin, type II cytoskeletal 2 epidermal	959	65 678	8.07
P07724	Serum albumin	878	68 693	5.75
P04259	Keratin, type II cytoskeletal 6B	716	60 315	8.09
P13647	Keratin, type II cytoskeletal 5	546	62 568	7.58
Q6IFZ6	Keratin, type II cytoskeletal 1b	374	61 379	7.73
P01870	Ig gamma chain C region	220	35 404	8.62
P20768	Ig mu chain C region	190	50 727	5.81
Q6P0K8	Junction plakoglobin	188	81 801	5.75

### IgM in bCM 1 inhibited P protein–HBGA interactions

**Fig. 1:**
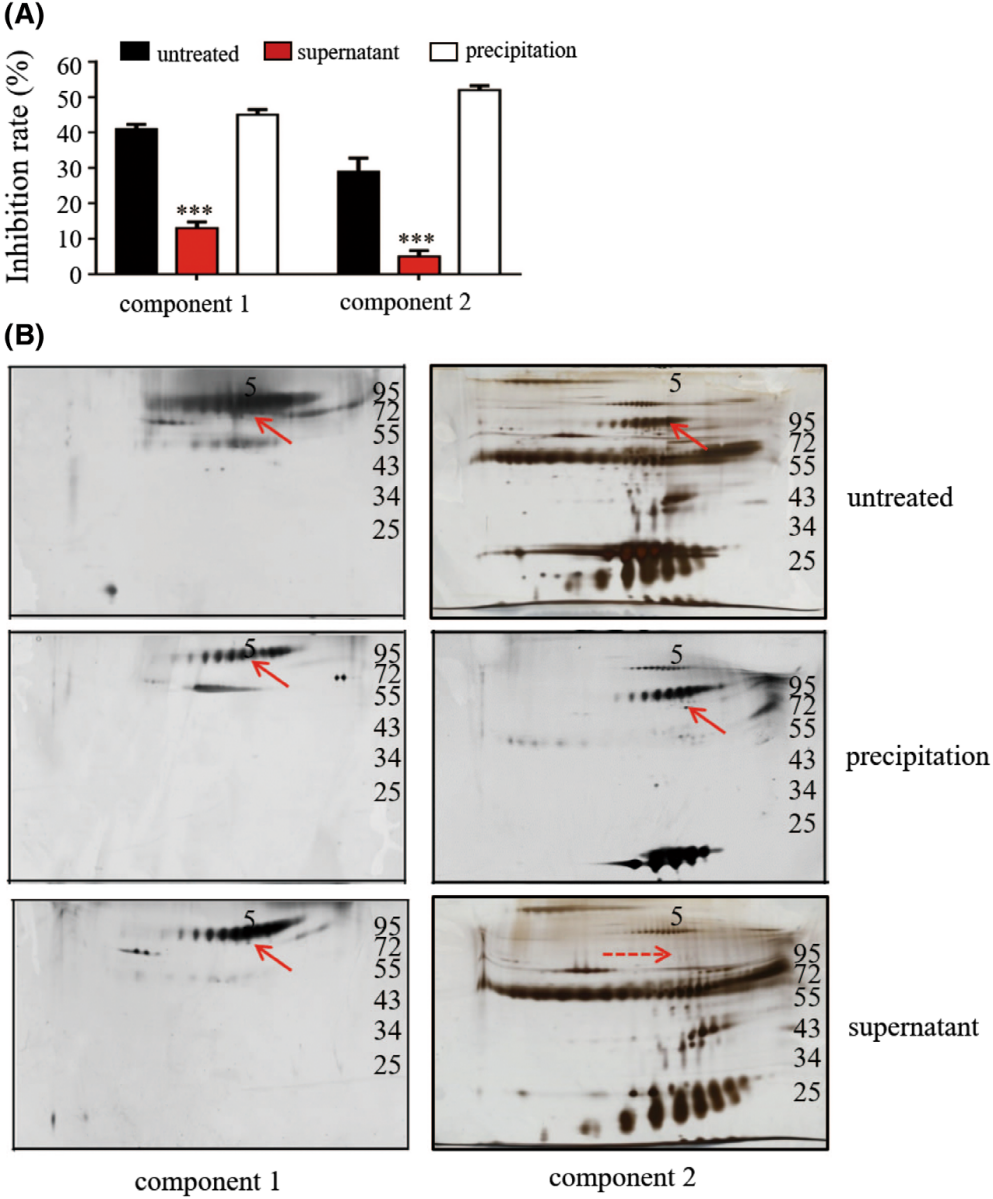
Identification of bovine IgM as the functional protein that inhibits HuNoV P protein–histo‐blood group antigen (HBGA) interactions. (A and B) Two separate components (component 1 and component 2) of bovine colostrum rich in the target protein were treated with water dialysis, followed by recovery of the supernatant and precipitation fractions. The recovered fractions were then analyzed for blocking effects against GII.4 HuNoV P protein–HBGA interactions by ELISA (A). The two components were analyzed by two‐dimensional‐polyacrylamide gel electrophoresis (B). Arrows indicate the IgM spots that do not occur in the supernatant of component 2 (dotted arrow). The data are presented as the mean ± SD values, Student's *t*‐test; ***, *P* < 0.001. All experiments were repeated at least three times. [Colour figure can be viewed at wileyonlinelibrary.com]

IgM belongs to the euglobulin family and has very low solubility in pure water. The two components of bCM1 were dialyzed against ultrapure water at 4 °C for 3 h, and the supernatant and precipitate (resuspended in distilled water) were recovered separately. The untreated samples, supernatants, and precipitates were tested for their ability to block P protein–HBGA interactions using the ELISA‐based blocking assay [21]. The results showed that the inhibition ability of the precipitates was the strongest, whereas that of the supernatants was greatly reduced, indicating that the functional proteins were indeed precipitated (Fig. 1A). The proteins in the two components were separated using 2D‐PAGE analysis. As indicated by the arrow in Fig. 1B, IgM precipitated after water dialysis. Owing to the low total protein concentration of component 1, only approximately half of the IgM was precipitated by dialysis treatment. Interestingly, the two sets of precipitates shared a common IgM protein, even though IgM could hardly be detected in the supernatant of component 2, which showed only marginal inhibitory ability. These data indicated a correlation between the occurrence of IgM and its inhibitory ability. Based on these results, we concluded that IgM was most likely the major functional protein in bCM that inhibits P protein–HBGA interactions.

### IgM as the functional protein in bovine colostrum samples

**Fig. 2:**
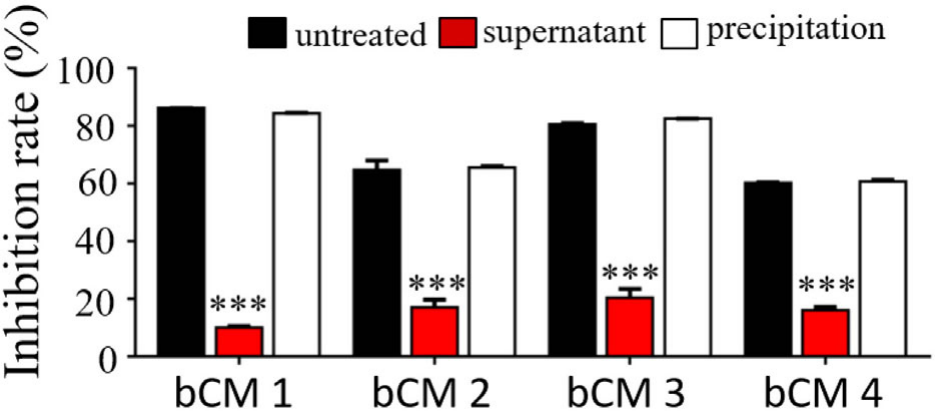
Identification of IgM in bovine colostrum samples as the functional protein that inhibits HuNoV P protein–histo‐blood group antigen (HBGA) interactions. Four bovine colostrum samples were treated with water dialysis, followed by recovery of the supernatant and precipitation fractions. The recovered fractions were then analyzed by ELISA for blocking effects against GII.4 HuNoV P protein–HBGA interactions. The data are presented as the mean ± SD values, Student's *t*‐test; ***, *P* < 0.001. bCM 1, bCM sample 1; bCM 2, bCM sample 2; bCM 3, bCM sample 3; bCM 4, bCM sample 4. All experiments were repeated at least three times. [Colour figure can be viewed at wileyonlinelibrary.com]

To further verify that IgM is the functional protein, as opposed to any other mature antibodies of non‐IgM molecules, IgM and other antibodies were preliminarily separated from all colostrum samples by dialysis and centrifugation by taking advantage of the precipitation features of IgM in pure water. The precipitated IgM was dissolved in distilled water, and the distribution of inhibition ability in different dialysis components was detected using a receptor‐binding inhibition experiment. The results showed that all colostrum samples behaved similarly to bCM1 after dialysis treatment in terms of inhibiting P protein–HBGA interactions. The inhibition ability of the precipitate was almost the same as that of the original sample, whereas the inhibition ability of the supernatant remained very weak. These data support that the functional proteins inhibiting P protein–HBGA interactions in all colostrum samples were IgM antibodies, as in bCM1, which were mainly present in the precipitate (Fig. 2).

### Inhibition of P protein–HBGA interactions weakened after removal of IgM

**Fig. 3:**
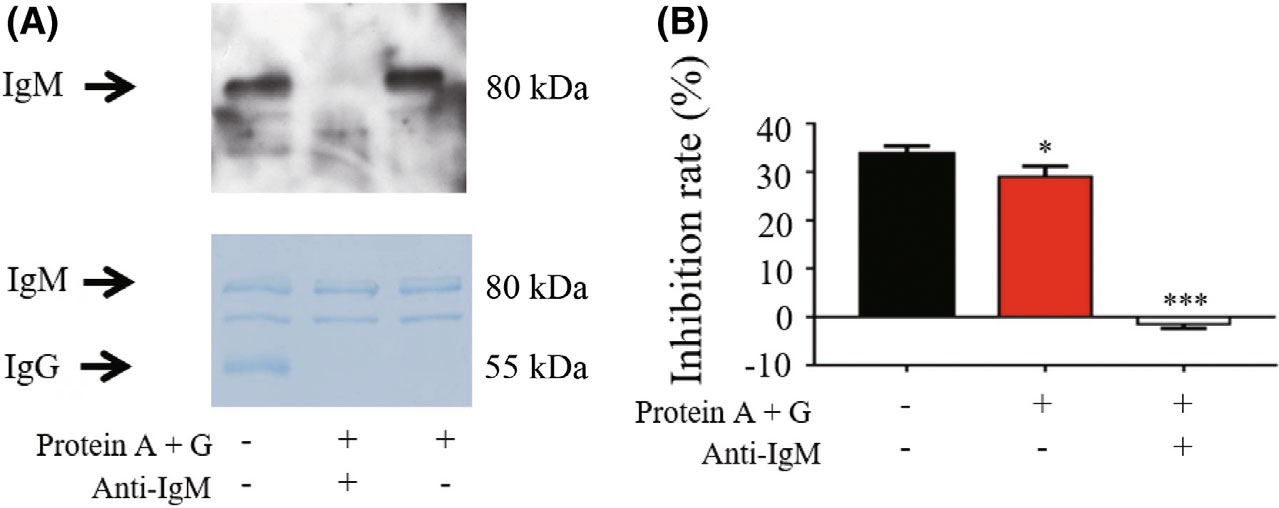
Bovine colostrum (bCM) sample lacking IgM showed significantly reduced blockage abilities against HuNoV P protein–histo‐blood group antigen (HBGA) interactions. (A and B) The IgM of bCM was specifically removed by co‐immunoprecipitation. The IgM was detected by western blot and Coomassie brilliant blue staining (A). Blocking effects of IgM and non‐IgM fractions of bCM against GII.4 HuNoV P protein–HBGA interactions were detected by ELISA (B). The data are presented as the mean ± SD values, Student's *t*‐test; *, *P* < 0.05; ***, *P* < 0.001. All experiments were repeated at least three times. [Colour figure can be viewed at wileyonlinelibrary.com]

To further verify IgM as the inhibitory factor of bCM, the IgM component was removed from bCM using co‐immunoprecipitation (Co‐IP). We showed that an anti‐bovine IgM antibody immobilized with Protein A + G could remove IgM from the bCM nearly completely, as shown by western blotting analysis that could not be detected IgM after removal (Fig. 3A up). Coomassie brilliant blue staining showed that in the two bCM samples treated with Protein A + G, all IgM was removed; however, due to the coincidence of IgM, lactoferrin (78 056 Da), polymeric immunoglobulin receptor (pIgR; 82 435 Da), and other electrophoresis positions, only the staining diagram could not observe the difference in IgM disposal (Fig. 3A down). As expected, the treated bCM showed a significantly reduced ability to block P protein–HBGA interactions compared with that of the untreated control bCM (Fig. 3B). These data confirmed that the functional protein in bCM that inhibited HuNoV P protein–HBGA interactions was IgM.

### Pure IgM inhibited P protein–HBGA interactions

**Fig. 4:**
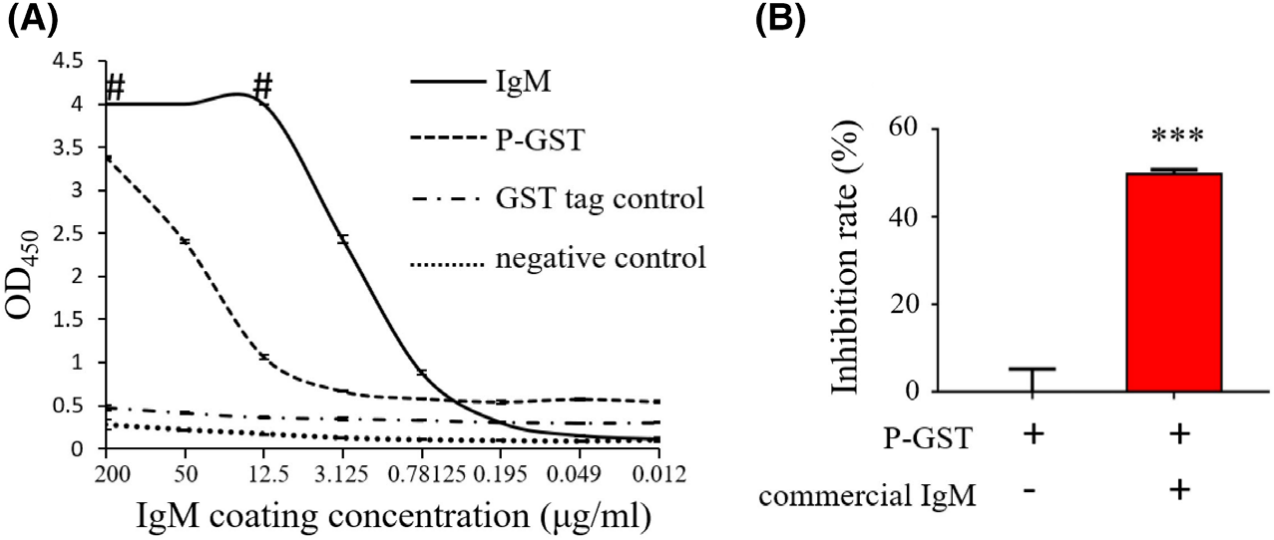
Pure IgM inhibiting GII.4 HuNoV P protein–histo‐blood group antigen (HBGA) interactions. (A) Serial diluted bovine IgM was coated to the wells of microtiter plates. The coated IgM was detected by rabbit anti‐IgM antibody. P‐GST fusion protein (5 μg·mL^−1^) and GST (21 μg·mL^−1^) were incubated with coated IgM after being blocked with 5% nonfat milk in PBST, and blocking buffer was used as the negative control. Bound P‐GST and GST proteins were detected by rabbit anti‐GST antibody. #, Optical density at 450 nm (OD_450_) outranged the capacity of the instrument. (B) Blocking effects of commercial IgM against GII.4 HuNoV P protein–HBGA interactions were detected by ELISA. The data are presented as the mean ± SD values, Student's *t*‐test; ***, *P* < 0.001. All experiments were repeated at least three times. [Colour figure can be viewed at wileyonlinelibrary.com]

With the confirmation of the inhibitory effect of IgM, we inferred that effective IgM is not produced by acquired immunity; instead, it is most likely innate (a natural antibody). If this is the case, IgM should be widely present in bovine and in the blood of fetal cows that have not been stimulated by external immunogens. Indeed, our data supported this conjecture, commercial pure bovine IgM bound the GII.4 HuNoV P protein, and inhibited its interaction with HBGAs, verifying that pure bovine IgM possessed an inhibitory ability (Fig. 4).

### Fetal bovine serum (FBS) inhibits P protein–HBGA interactions

**Fig. 5:**
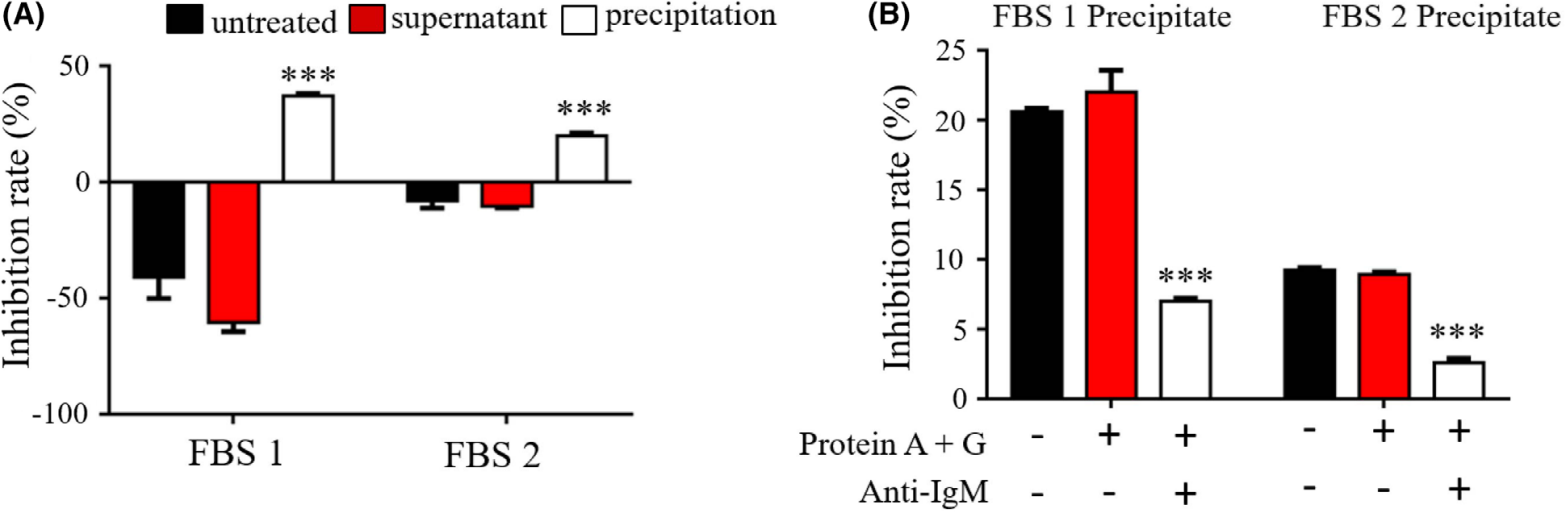
Fetal bovine serum (FBS) could inhibit GII.4 HuNoV P protein–HBGA interactions. (A) FBS samples were dialyzed with water; supernatants and precipitates were collected, and their inhibition rates were measured by ELISA. (B) IgM in FBS precipitates was specifically removed by co‐immunoprecipitation, and the blocking effects of IgM and non‐IgM fractions of FBS P against GII.4 HuNoV P protein–HBGA interactions were detected by ELISA. The data are presented as the mean ± SD values, Student's *t*‐test; ***, *P* < 0.001. All experiments were repeated at least three times. [Colour figure can be viewed at wileyonlinelibrary.com]

Natural antibodies begin to appear in the embryonic period, mature in the infant period, and exist conservatively throughout life, and the type of antibodies present does not change. If the detected bCM IgM is a natural antibody, the same antibody may also be present in fetal bovine serum (FBS). To prove this, water dialysis was used to treat two FBS samples from two different commercial brands, and the IgM in the samples was separated from other serum proteins. The untreated FBS, supernatants, and precipitates were tested for their ability to block P protein–HBGA interactions. The results were completely consistent with the initial hypothesis that the FBS from both different brands could inhibit P protein–HBGA interactions, and this ability was the same as that in bCM, which existed in the precipitation components after water dialysis treatment (Fig. 5A). It was also noted that untreated FBS did not show inhibitory effects against P protein–HBGA interactions, which was consistent with previous reports that natural IgM (nIgM) in serum was generally bound by autoantigens, and the interaction between nIgM and non‐autoantigens in the serum environment would be inhibited [28]. Protein A + G and anti‐IgM antibodies were used to specifically remove IgM from the highly inhibitory components. Finally, as expected, after co‐IP to specifically remove IgM, the inhibitory ability of the precipitate was significantly reduced (Fig. 5B). Taken together, we concluded that cattle have natural IgM antibodies that can block GII.4 HuNoV P protein–HBGA interactions.

## Discussion

HuNoV is the main cause of acute nonbacterial gastroenteritis in humans. The vast majority of HuNoV infections are caused by GI and GII, with GII being more prevalent than GI, causing more than 80% of all HuNoV infections [29, 30, 31]. GII.4 viruses alone can cause up to 90% of HuNoV infections during high outbreak seasons when a new variant first appears [3, 29]. Due to the lacking of an effective conventional culture system and a useful small animal model, the mechanisms behind HuNoV infection and pathogenesis are not fully understood. For patients who are already infected and need medical help, the main focus is on targeted treatment to relieve symptoms, such as hydration and electrolytes, attention to prevent secondary infection, and rest to recover strength. Main countermeasures to prevent HuNoV infection include to pay attention to personal hygiene, not to contact with obviously infected people, and not to dine in public places during the outbreak period to reduce the risk of being infected [32]. In this study, we found that bovine colostrum sample 1 (bCM1) can inhibit GII.4 HuNoV P protein–HBGA interactions and further demonstrated that the functional component in bCM1 is IgM. This finding provides a research basis for further development of IgM into an antiviral drug against HuNoVs.

Numerous studies have shown that bCM is high in nutritional value, growth factors, and hormones that promote the growth and development of infants and newborn animals [33]. In addition, more than 40 immune‐related proteins [34] and cells, including neutrophils, macrophages, and lymphocytes [15], are present in bCM, providing innate and acquired immune protection for pups. bCM contains a variety of antibodies, and IgG1 represents the largest proportion of the total antibodies, accounting for 75%, followed by IgM, IgA, and IgG2. Numerous studies have shown that bovine IgG broadly inhibits pathogenic microorganisms that infect humans, such as *Streptococcus pyogenes* that causes dental caries, *Candida albicans* that causes deep infections, *Cryptosporidium*, *Escherichia coli*, and *Helicobacter pylori* that causes gastroenteritis [35, 36]. However, our preliminary study found that IgG contained in bCM did not inhibit the interaction of GII.4 HuNoV P protein with HBGA [21]. IgM is the second most abundant antibody in bCM, and although it is lower than IgG, its ability to activate complement responses, inhibit pathogen‐receptor binding, agglutinate pathogens, and neutralize toxins and viruses is stronger than that of IgG [24]. It is effective in neutralizing and destroying viruses [25] such as SARS‐COV‐2 [26] and mucosal HIV [27]. In this study, four bCM samples, pure IgM and two FBS samples were found to block the P protein of the HuNoV receptor‐binding domain interacting with its HBGA receptors, implying that the bCMs have anti‐HuNoV effects. Our data in this study further showed that the functional protein in bCM is a natural antibody IgM.

The production of natural antibodies does not require stimulation by external antigens and generally occurs during natural development [37]. The recognition profile is conserved within the same species and is independent of external stimuli [37]. IgM is the most important natural antibody. Natural antibodies have two important characteristics, the ability to bind with low affinity to antigens and the ability to bind to multiple antigens. The main difference from conventional antibodies is that conventional antibodies specifically bind a high‐affinity antigen, whereas natural antibodies can gently recognize a class of structurally similar antigens [24, 38]. A large number of bacteria and viruses have been identified that can be recognized by natural antibodies, such as *Streptococcus pneumoniae*, *E. coli BL21* [39], *Thermite tularex* [40], and *influenza virus* [38]. In the present study, the natural antibody IgM was found to inhibit the binding of the GII.4 HuNoV P domain to its receptors. The binding ability of IgM to GII.4 HuNoV P protein was weak and significantly influenced by the solubility of the solution, and both features are in line with the characteristics of natural antibodies. More importantly, the natural antibody IgM is commonly found in serum, so the bovine natural antibody IgM with anti‐HuNoV effects may be widely available and beneficial for practical production and application.

On the contrary, in our previous study, we found that bCM blocks VLPs of two genotypes (GII.4 and GII.9) of HuNoVs binding to their viral receptors [19], therefore speculate that the ant‐HuNoV effects of IgM may be general and may not be HuNoV genotype dependent. However, because of the lack of one validated protocol/equipment to produce and then verify the particle or VLP, at the initial stage, we chose this published alternative method with *E. coli* expressed the P‐GST [13], after confirmed that this P‐GST could specifically bind with HBGA through virus P protein rather than GST tag, we proceed our focus to the identification and verification of the inhibitory component of bCM. We believe this submitted work is just the beginning and more experiments are needed for further study in future.

## Conclusions

In summary, this study found that the natural antibody IgM in bovine colostrum binds HuNoV P protein, thereby inhibiting its binding to HBGA receptor and potentially blocking HuNoV infection. Our new findings provide a basis for potential application of bovine IgM in the prevention and treatment of HuNoV infection.

## Conflict of interest

The authors declare no conflict of interest.

## Author contributions

Author contributions are as follows. QH, ZX, LW, and HC contributed to data curation, formal analysis and methodology; MT and RZ contributed to conceptualization; QH and RZ contributed to writing—original manuscript; MT contributed to revision. All authors have read and agreed to the published version of the manuscript.

## Data Availability

The data that support the findings of this study are available in this article.
